# Gene therapy for lysosomal storage diseases: Current clinical trial prospects

**DOI:** 10.3389/fgene.2023.1064924

**Published:** 2023-01-13

**Authors:** Jun Kido, Keishin Sugawara, Kimitoshi Nakamura

**Affiliations:** ^1^ Department of Pediatrics, Faculty of Life Sciences, Kumamoto University, Kumamoto, Japan; ^2^ Department of Pediatrics, Kumamoto University Hospital, Kumamoto, Japan

**Keywords:** adeno-associated viral vector, clinical trial, gene therapy, lentiviral vector, lysosomal storage disease

## Abstract

Lysosomal storage diseases (LSDs) are a group of metabolic inborn errors caused by defective enzymes in the lysosome, resulting in the accumulation of undegraded substrates. LSDs are progressive diseases that exhibit variable rates of progression depending on the disease and the patient. The availability of effective treatment options, including substrate reduction therapy, pharmacological chaperone therapy, enzyme replacement therapy, and bone marrow transplantation, has increased survival time and improved the quality of life in many patients with LSDs. However, these therapies are not sufficiently effective, especially against central nerve system abnormalities and corresponding neurological and psychiatric symptoms because of the blood-brain barrier that prevents the entry of drugs into the brain or limiting features of specific treatments. Gene therapy is a promising tool for the treatment of neurological pathologies associated with LSDs. Here, we review the current state of gene therapy for several LSDs for which clinical trials have been conducted or are planned. Several clinical trials using gene therapy for LSDs are underway as phase 1/2 studies; no adverse events have not been reported in most of these studies. The administration of viral vectors has achieved good therapeutic outcomes in animal models of LSDs, and subsequent human clinical trials are expected to promote the practical application of gene therapy for LSDs.

## 1 Introduction

Lysosomal storage diseases (LSDs) are a group of progressive inherited metabolic diseases caused by impaired lysosomal functions due to factors such as defects in lysosomal enzymes and transporters that may result in the overaccumulation of undegraded substrates. Patients with LSDs present with a broad spectrum of clinical manifestations owing to the wide impact of the accumulated substrates and the diverse sites of accumulation. To date, more than 50 different LSDs have been identified based on different causative enzymes. The incidence of each LSD is rare, and the combined incidence is estimated to be 1 per 7,000–9,000 live births (Fletcher, 2006).

LSDs are progressive diseases; the rate of progression varies with each disease and patient. In recent years, the availability of effective treatment options, including substrate reduction therapy, pharmacological chaperone therapy, enzyme replacement therapy (ERT), and bone marrow transplantation, has increased survival time and improved the quality of life of many patients with LSDs (Beck, 2007; Solomon and Muro, 2017; de Oliveira Poswar et al., 2019). However, most applications of ERT, except for IZCARGO^®^ (Pabinafusp Alfa), do not affect the brain because the drugs cannot cross the blood-brain barrier (BBB) (Okuyama et al., 2021). Substrate reduction therapy has limited effects on neurological manifestations, even if the compounds cross the BBB. Bone marrow transplant, if performed timely before the development of definite neurological manifestations, may prevent the progression of neurological manifestations in some LSDs. Although the mucopolysaccharidosis (MPS) type I (or Hurler Syndrome), in which hematopoietic stem cell (HSC) transplantation has been widely applied in the last decades, obtaining quite positive results, the other MPSs, mainly MPS type II, showed no evidenced efficacy of HSC transplantation in the brain impairment of the children, except in very rare case. Therefore, while some of these conventional therapies may have limited effects on the brain of patients with LSDs, they do not offer a definitive treatment for the neurological pathology associated with LSDs.

With ongoing development, gene therapy is expected to become a promising therapy for the neurological pathology of LSDs (Rastall and Amalfitano, 2015; Penati et al., 2017; Poswar et al., 2017; Beck, 2018). Herein, we present the current state of LSD-related gene therapies for which clinical trials have been conducted, are currently being conducted or will be conducted in the near future. Furthermore, we discuss the role and future prospects of clinical gene therapy applications.

## 2 Gene therapy in LSD: Clinical trials and literature review

We searched for clinical trials of gene therapy for LSDs using the ClinicalTrials.gov public database (https://clinicaltrials.gov/ct2/home). Within this database, we entered names of LSDs such as “Fabry disease” for “Condition or disease” and “gene therapy” for “Other terms.” Moreover, we surveyed reports of gene therapy for LSDs available in PubMed (https://pubmed.ncbi.nlm.nih.gov) or Google Scholar (https://scholar.google.com) using keywords such as “lysosomal storage disease,” “Fabry disease,” and “gene therapy.” These searches yielded clinical trials of gene therapy for LSDs that are ongoing or have been completed and are summarized in Table 1. Gene therapy studies conducted in model animals with LSDs, whose clinical trials in humans have not been conducted, are summarized in Table 2.

**TABLE 1:** Gene therapy-based clinical trials for lysosomal storage diseases.

Disease	NCT number	Sponsor (code name)	Status (accessed April 2022)	*n* (age/sex)	Vector (carrying gene), administration, effects, and adverse events	References
Fabry	04040049	Freeline Therapeutics (FLT190)	Ph 1/2, Recruiting	15 (>18 years/M)	AAVS3 (*GLA*), Intravenous	Hughes et al. (2020)
	04455230		Recruiting	50 (>18 years/M)		
	04046224	Sangamo Therapeutics (ST-920)	Ph 1/2, Recruiting	48 (>18 years/M and F)	AAV2/6 (*GLA*), Intravenous	Yasuda et al. (2020), Hughes et al. (2019)
	03454893	AvroBio (AVR-RD-01)	Ph 1/2, Terminated	11 (16 years–50 years/M)	LV (*GLA*), *Ex vivo*	
	04999059		LTF, Enrolling by invitation	11 (16 years–50 years/M)		
	02800070	Ozmosis Research Inc.	Ph 1, Active, not recruiting	5 (48 years/M, 39 years/M, 39 years/M, 37 years/M, 29 years/M)	LV (*GLA*), *Ex vivo*	Khan et al. (2021)
					Almost normal GLA activity levels within 1 week. No serious adverse events.	
	04519749	4D Molecular Therapeutics (4D-310)	Ph 1/2, Recruiting	18 (>18 years/M)	AAV (*GLA*), Intravenous	
Pompe	04093349	Spark Therapeutics (SPK-3006)	Ph 1/2, Recruiting	30 (>18 years/M and F)	AAV (*GAA*), Intravenous	
	04174105	Audentes Therapeutics (AT845)	Ph 1/2, Recruiting	12 (18 years–80 years/M and F)	AAV8 (*GAA*), Intravenous	
	00976352	University of Florida	Ph1/2, Completed	96 m/M, 108 m/M, 66 m/M, 180 m/M, 30 m/F	AAV1 (*GAA*), Intra diaphragm	Smith et al. (2013)
					Safe and moderate improvements in volitional ventilatory performance.	
					Expected levels of immune responses.	
	02240407		Ph1, Completed	2 (18 years–50 years/M and F)	AAV9 (*GAA*), Intramuscular	
	03533673	Asklepios Biopharmaceutical, Inc. (ACTUS-101)	Ph 1/2, Recruiting	8 (>18 years/M and F)	AAV2/8 (*GAA*), Intravenous	Kishnani and Koeberi (2019)
Gaucher	00001234	NINDS	Completed	3 (22 years/M, 22 years/F, 21 years/M)	RV (*GBA* + *GLA*), *Ex vivo*	Dunbar et al. (1998)
					No serious adverse events	
	04145037	AvroBio (AVR-RD-02)	Ph 1/2, Recruiting	16 (18 years–50 years/M and F)	LV (*GBA*), *Ex vivo*	Dahl et al. (2020)
	04836377		LTF, Enrolling by invitation	16 (18 years–50 years/M and F)		
	04411654	Prevail Therapeutics (PR001)	Ph 1/2, Recruiting	15 (<24 m/M and F)	AAV9 (*GBA*), Intracisternal	
	05324943	Freeline Therapeutics (FLT201)	Ph 1/2, Recruiting	18 (>18 years/M and F)	AAVS3 (*GBA*), Intravenous	
Krabbe	04771416	Passage Bio, Inc. (PBKR03)	Ph 1/2, Recruiting	24 (1 m–9 m/M and F)	AAVHu68 (*GALC*), Intracisternal	Hordeaux et al. (2022)
	04693598	Forge Biologics, Inc. (FBX-101)	Ph 1/2, Active, not recruiting	6 (<12 m/M and F)	AAVrh10 (*GALC*), Intravenous after HSCT	Bradbury et al. (2018)
MPS I	02702115	Sangamo Therapeutics (SB-318)	Ph 1/2, Terminated	3 (>5 years/M and F)	AAV2/6 (ZFN-mediated genome editing), Intravenous, SB-318 had a favorable safety profile and with evidence of targeted genome editing in liver up to 48 weeks after exposure.	Harmatz et al. (2022), Ou et al. (2020)
	04628871		LTF, Enrolling by invitation	3 (>5 years/M and F)		
	03580083	Regenxbio Inc. (RGX-111)	Ph 1/2, Recruiting	11 (>4 m/M and F)	AAV9 (*IDUA*), Intracisternal	
	03488394	IRCCS San Raffaele	Ph 1/2, Active, not recruiting	8 (1.97 years/M, 1.15 years/M, 1.95 years/F, 1.12 years/M, 2.84 years/M, 2.13 years/M, 1.71 years/M, 1.97 years/F)	LV (*IDUA*), *Ex vivo*	Gentner et al. (2021)
					Increased IDUA activity in blood and cerebrospinal fluid, decreased urinary glycosaminoglycan excretion, stable cognitive performance, stable motor skills, improved or stable findings on MRI of the brain and spine, reduced joint stiffness, and normal growth.	
MPS II	00004454	University of Minnesota	Ph 1/2, Completed	2 (>18 years/M and F)	RV-L2SN (*IDS*), *Ex vivo*	
	04571970	Regenxbio Inc. (RGX-121)	Ph 1/2, Recruiting	6 (5 years–17 years/M)	AAV9 (*IDS*), Intracisternal	
	03566043		Ph 1/2, Recruiting	18 (4 m–5 years/M)		
	04597385		LTF, Enrolling by invitation	12 (>28 m/M)		
	03041324	Sangamo Therapeutics (SB-913)	Ph1/2, Terminated	9 (>5 years/M and F)	AAV2/6 (ZFN-mediated genome editing), Intravenous, SB-913 had a favorable safety profile and with evidence of targeted genome editing in liver up to 48 weeks after exposure.	Harmatz et al. (2022)
	04628871		LTF, Enrolling by invitation	9 (>5 years/M and F)		
	05238324	Homology Medicines, Inc. (HMI-203)	Ph 1, Recruiting	9 (18 years–30 years/M)	AAV (*IDS*), Intravenous	
MPS IIIA	01474343	LYSOGENE (LYS-SAF301)	Ph1/2, Completed	4 (6 years 7 m/F, 6 years 5 m/M, 5 years 10 m/F, 2 years 8 m/M)	AAVrh10 (*SGSH* + *SUMF1*), Intracisternal	Tardieu et al. (2014)
					Moderate improvement in behavior, attention, and sleep. Younger patient likely to display neurocognitive benefit.	
					No serious adverse events	
	02053064	LYSOGENE (LYS-SAF302)	Ph1/2, Completed	4 (>6 m/M and F)	AAVrh10 (*SGSH*), Intracisternal	
	03612869		Ph2/3, Active, not recruiting	20 (>6 m/M and F)		
	2015–000359–26^a^	Laboratorios del Dr. Esteve, S.A. (EGT-101)	Ph 1/2, on going	6 (<18 years/M and F)	AAV9 (*SGSH*), Intracerebroventricular administration	
	04088734	Abeona Therapeutics, Inc. (ABO-102)	Ph 1/2, Terminated	5 (>6 m/M and F)	AAV9 (*SGSH*), Peripheral limb vein	Fu et al. (2016)
	02716246		Ph 1/2, Recruiting	22 (>6 m/M and F)		
	04360265		LTF, recruiting	50 (>6 m/M and F)		
	04201405	University of Manchester (OTL-201)	Ph1/2, Active, not recruiting	5 (3 m–24 m/M and F)	LV (*SGSH*), *Ex vivo*	Ellison et al. (2019)
MPS IIIB	03300453	UniQure Biopharma B.V. (AMT-110)	Ph1/2, Completed	4 (20 m/F, 26 m/M, 30 m/M, 53 m/M)	AAV2/5 (*NAGLU*), Intracisternal	Tardieu et al. (2017), Gougeon et al. (2021)
					Stable CNS transgene expression 66 months after surgery. Improved neurocognitive development in all patients, with the youngest patient having function close to that in healthy children.	
	03315182	Abeona Therapeutics, Inc. (ABO-101)	Ph1/2, Active, not recruiting	15 (>6 m/M and F)	AAV9 (*NAGLU* + CMV enh), Peripheral limb vein	Ribera et al. (2015)
	04655911		LTF, recruiting	24 (>6 m/M and F)	AAV9 (*NAGLU*), Peripheral limb vein	
MPS VI	03173521	Fondazione Telethon	Ph1/2, Active, not recruiting	9 (4 years–65 years/M and F)	AAV2/8 (*ARSB*), Intravenous	Ferla et al. (2017)
GM1 gangliosidosis	03952637	NHGRI (AXO-AAV-GM1)	Ph 1/2, Recruiting	45 (6 m–12 m/M and F)	AAV9 (*GLB1*), Intravenous	Latour et al. (2019)
	04273269	LYSOGENE (LYS-GM101)	Ph 1/2, Recruiting	16 (<3 years/M and F)	AAVrh10 (*GLB1*), Intracisternal	
	04713475	Passage Bio, Inc. (PBGM01)	Ph 1/2, Recruiting	20 (4 m–36 m/M and F)	AAVrh68 (*GLB1*), Cisterna magna	
Tay-Sachs/Sandhoff	04669535	Sio Gene Therapies (AXO-AAV-GM2)	Ph 1, Recruiting	18 (6 m–12 years/M and F)	AAV8 (*HEXA*) + AAV8 (*HEXB*), Intracisternal/Intrathecal	Flotte et al. (2022)
	04798235	Taysha Gene Therapies, Inc. (TSHA-101)	Ph 1/2, Active, not recruiting	6 (<15 m/M and F)	AAV9 (*HEXA* + *HEXB*), Intrathecal	
Cystinosis	03897361	AvroBio (AVR-RD-04/CTNS-RD-04)	Ph 1/2, Recruiting	6 (>18 years/M and F)	LV (*CTNS*), *Ex vivo*	Harrison et al. (2013)
	05146830		LTF, Not yet recruiting	50 (14 years–50 years/M and F)		
Metachromatic leukodystrophy	04283227	Orchard Therapeutics (OTL-200)	Ph3, Recruiting	6 (>6 m/M and F)	LV (*ARSA*), *Ex vivo*	
	03392987		Ph2, Active, not recruiting	10 (<6 years/M and F)		
	01560182	Approved as Libmeldy^Ⓡ^ at Dec. 2020 by EMA.	Ph1/2, Active, not recruiting	Late infantile: n = 16 (M = 10, F = 6, mean age 12.8 ± 4.3 m)	LV (*ARSA*), *Ex vivo*	Sessa et al. (2016), Fumagalli et al. (2022)
				Early juvenile: n = 13 (M = 6, F = 7, mean age 65.9 ± 33.4 m)	Prevention of disease onset or halted disease progression.	
	01801709	Institut National de la Santé Et de la Recherche Médicale	Ph1/2, Active, not recruiting	5 (6 m–5 years/M and F)	AAV10 (*ARSA*), Intracranial	Piguet et al. (2012)
	02559830	Shenzhen Second People’s Hospital	Ph 1/2, Recruiting	50 (2 years–45 years/M and F)	LV (*ARSA* + *ABCD1*), *Ex vivo*	
	03725670	Shenzhen Geno-Immune Medical Institute	Unknown	10 (>1 m/M and F)	LV (*ARSA*), *Ex vivo*	
LINCL	02725580	Amicus Therapeutics (AT-GTX-501)	Ph1/2, Completed	13 (>1 year/M and F)	AAV9 (*CLN6*), Intrathecal	
	04273243		LTF, Active, not recruiting	10 (>1 year/M and F)		
	01414985	Weill Medical College of Cornell University	Ph1/2, Completed	8 (3 years–18 years/M and F)	AAV10 (*CLN2*), Intracerebral	
	01161576		Ph1, Completed	12 (2 years–18 years/M and F)		
	00151216		Ph1, Completed	10 (8.6 years/M, 10 years/M, 6.9 years/M, 8.1 y/F, 4.5 years/F, 5.4 years/M, 4.5 years/M, 3.6 years/F, 5.3 years/M, 3.4 years/F)	AAV2 (*CLN2*), Intracranial	Worgall et al. (2008)
					A significantly reduced rate of neurological decline.	
CLN3	03770572	Amicus Therapeutics (AT-GTX-502)	Ph1/2, Active, not recruiting	7 (3 years–10 years/M and F)	AAV9 (*CLN3*), Intrathecal	
CLN5	05228145	Neurogene Inc. (NGN-101)	Ph 1/2, Recruiting	3 (3 years–8 years/M and F)	AAV9 (*CLN5*), Intracerebroventricular and intravitreal	
CLN7	04737460	Benjamin Greenberg	Ph 1, Recruiting	4 (1 year–18 years/M and F)	AAV9 (*CLN7*), Intrathecal	
Danon disease	03882437	Rocket Pharmaceuticals Inc. (RP-A501)	Ph 1, Recruiting	7–10 (>8 years/M)	AAV9 (*LAMP2B*), Intravenous	

^a^ EudraCT number, AAV, adeno-associated virus; ABCD, adenosine-triphosphate-binding cassette, sub-family D; ARSA, arylsulfatase A; ARSB, arylsulfatase B; CLN, ceroid-lipofuscinosis neuronal protein; CTNS, transmembrane lysosomal cystine transporter—cystinosin; GALC, galactosylceramidase; GBA, glucocerebrosidase; GLA, α-galactosidase A; HEXA, β-hexosaminidase A alpha subunit; HEXB, β-hexosaminidase A beta subunit; HSCT, hematopoietic stem cell transplantation; IDS, iduronate-2-sulfatase; IDUA, α-L-iduronidase; LTF, long-term follow-up; LINCL, late-infantile neuronal ceroid lipofuscinosis; LV, lentivirus; RV, retrovirus; NAGLU, Aalpha-N-acetylglucosaminidase; NCL, neuronal ceroid lipofuscinoses; NHGRI, national human genome research institute; NINDS, national institute of neurological disorders and stroke; SGSH, N-sulfoglucosamine sulfohydrolase; SUMF1, sulfatase modifying factor 1; ZFN, zinc finger nuclease. References shown in bold font report the results of clinical trials.

**TABLE 2:** Gene therapy-based pre-clinical studies for lysosomal storage diseases.

Disease	Model (administration)	Vector (carrying gene)	References
MPS IIID	*Gns* ^−/−^ mice (cisterna magna)	AAV9 (*Gns*)	Roca et al. (2017)
MPS IVA	KO-mice (intravenous)	AAV8 (*GALNS*)	Sawamoto et al. (2020)
MPS VII	*Gus* ^mps/mps^ mice (superficial temporal vein)	LV (*Gus*)	Macsai et al. (2012)
NCL (CLN1)	*Ppt1* ^−/−^ mice (intra-brain-parenchymal and/or intrathecal)	AAV2/9 (*PPT1*)	Shyng et al. (2017)
NCL (CLN8)	*Cln8* ^*mnd*^ (intracerebroventricular)	AAV8 (*CLN8*)	Johnson et al. (2021)
NCL (CLN10)	CtsD^−/−^ mice (intra-brain-parenchymal and/or liver/stomach)	AAV2 (CtsD)	Shevtsova et al. (2010)
Aspartylglucosaminuria	*Aga* ^−/−^ mice (intravenous)	AAV9 (*AGA*)	Chen et al. (2021)
Niemann-Pick C	*Npc1* ^−/−^ mice (left lateral ventricle and cisterna magna)	AAV9/3 (*NPC1*)	Kurokawa et al. (2021)
	*Npc1* ^−/−^ mice (intravenous)	Plasmid DNA (*NPC1*)	Jiang et al. (2020)
Alfa mannosidosis	AMD cat (intravenous)	AAVhu.32 (*fMANB*)	Yoon et al. (2020)
Galactosialidosis	*Ctsa* ^−/−^ mice (intravenous)	AAV2/8 (*CTSA*)	Hu et al. (2021)
Mucolipidosis IV	*Mcoln1* ^−/−^ mice (intravenous)	AAV9 (*MCOLN1*)	DeRosa et al. (2021)
Farber	*Asah1* ^P361R/P361R^ mice (temporal vein)	LV (*ASAH1*)	Alayoubi et al. (2013)

AMD, alpha-mannosidosis; fMANB, feline alpha-mannosidase gene; Gns, murine N-acetylglucosamine-6-sulfatase; Gus: murine β-glucuronidase gene; KO, knockout; LV, lentivirus; MoMLV, moloney murine leukemia virus; NCL, neuronal ceroid lipofuscinoses.

### 2.1 Fabry disease

**FIGURE 1:**
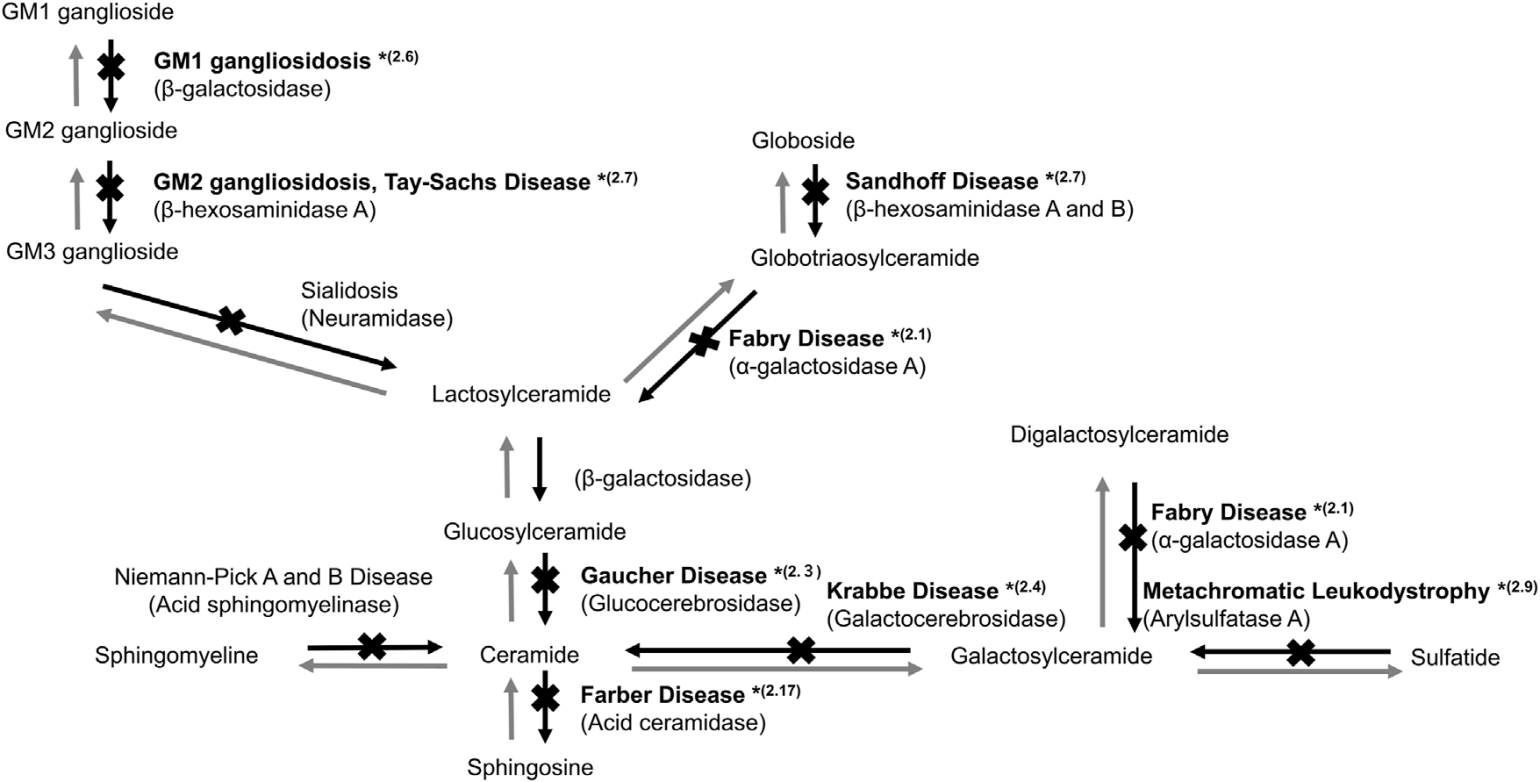
Sphingolipid metabolism in the lysosome. *(2.1, 2.3, 2.4, 2.6, 2.7, 2.9, and 2.17) indicate the sections in which studies on gene therapy for LSDs are described.

Fabry disease (FD, MIM number: 301500) is an X-linked LSD caused by a mutation in the *α*-galactosidase A (*GLA*) gene (Figure 1). FD is manifested by the progressive accumulation of glycosphingolipids, such as Gb3, especially in the blood vessels, heart, kidneys, brain, skin, and eyes. FD is divided into two types based on onset time and clinical manifestation. Patients with classic type FD present with hypohidrosis, acroparesthesias, angiokeratomas, and corneal opacities during childhood. Patients with late-onset FD develop cerebrovascular disease and cardiac and renal failure owing to Gb3 accumulation in the cardiac muscle and vascular endothelium.

FLT190 is a recombinant adeno-associated viral (AAV) vector containing human *GLA* cDNA. A single administration of FLT190 has the potential for continuous and durable expression of *GLA*. In *Gla* ^−/−^ mice, FLT190 administration induced hepatic *GLA* expression and durable levels of plasma GLA activity, which resulted in the uptake of GLA in the heart and kidney, clearance of inclusion bodies, a reduction in renal Gb3, and reduction in Gb3 and lyso-Gb3 levels in the plasma and other tissues. In the NCT 04040049 study, FLT190 was administered to 15 adult males, both treated and treatment-naive patients, with classic FD (Hughes et al., 2020). The long-term safety and durability of GLA in patients intravenously administered FLT190 were investigated (NCT 04455230) (Table 1).

ST-920 is a recombinant AAV2/6 vector encoding human *GLA* (Yasuda et al., 2020). In FD mice, a single dose of ST-920 increased GLA activity in the kidney, heart, liver, spleen, and plasma, and decreased Gb3 and lyso-Gb3 levels in the liver, heart, plasma, and kidney. A clinical trial on ST-920 is in progress; 48 FD patients (≥18 years old) have been infused intravenously with a single dose of ST-920 and their clinical course will be followed for 52 weeks (NCT 04046224) (Table 1) (Hughes et al., 2019).

AVR-RD-01 is an autologous drug product comprising a CD34+ cell-enriched fraction that contains cells transduced with a lentiviral (LV) vector encoding human *GLA*. A total of 20 classical FD male patients (≥16 years old), who had not previously received ERT and/or chaperone therapy within 3 years of the time of screening, received a one-time intravenous administration of AVR-RD-01 and were observed for 64 weeks. Moreover, a long-term follow-up study for 14 years of participants administered AVR-RD-01 is underway (NCT 04999059) (Table 1).

In the NCT 02800070 study, five male FD patients (18–50 years old) underwent autologous HSC transplantation using CD34+ cells transduced with LV containing human *GLA* cDNA and were observed for 5 years (Khan et al., 2021). All patients exhibited normal levels of GLA activity within 1 week of treatment and LV vector was detected in the peripheral blood and bone marrow cells. Analysis of plasma and leukocytes demonstrated an improvement in GLA activity to within or above the control reference range. Levels of plasma and urine Gb3 and lyso-Gb3 were also reduced. Three of the five patients could discontinue ERT (Table 1).

The recombinant AAV vector 4D-310 comprises two active components: A capsid (4D-C102) and a transgene cassette encoding human *GLA* driven by the CAG promoter. In the NCT 04519749 study, 18 male FD patients (≥18 years old) received a single intravenous administration of 4D-310 and were observed for 1 year (Table 1).

Liver-targeted (*in vivo*) and hematopoietic stem/progenitor cell (HSPC)-targeted (*ex vivo*) clinical trials for FD are ongoing. *In vivo* therapy involves the use of hepatotropic AAV vectors AAV2/6 and AAVS3 for the transfer of the *GLA* gene to hepatocytes and is expected to produce enzymes to express therapeutic effects. *Ex vivo* therapy involves the transduction of the *GLA* gene into patients’ CD34+ HSPCs using LV. The transduced HSPCs will deliver the functional enzyme to sites not accessible by ERT. In the future, the therapeutic strategy may be determined based on a patient’s disease type, severity, and target organ.

### 2.2 Pompe disease

Glycogen storage disease type II (MIM: 232300) known as Pompe disease (PD) is an autosomal recessive disease caused by a defect in *α*-glucosidase (AαGlu, EC: 3.2.1.20) encoded by *GAA*. PD manifests as the accumulation of lysosomal glycogen in the heart and skeletal muscles (Martiniuk et al., 1986); massive accumulation eventually leads to death from cardiorespiratory failure (Mellies and Lofaso, 2009). Patients with infantile-onset PD (IOPD), in which AαGlu activity is absent, present with hypertrophic cardiomyopathy and hypotonia, and often die within a few months after birth. Patients with late-onset PD (LOPD), in which AαGlu activity is reduced, present with skeletal muscle dysfunction, but rarely develop cardiac muscle manifestation. The onset time and severity of LOPD are variable, and patients predominantly manifest symptoms after 40 years of age (Hagemans et al., 2005).

SPK-3006 is an AAV vector encoding human *GAA.* The safety, tolerability, and efficacy of a single intravenous administration of SPK-3006 have been investigated in adults with moderate LOPD receiving ERT (NCT 04093349) (Table 1).

A study (NCT 04174105) using AT845, an AAV8 vector delivering human *GAA* cDNA under the control of a muscle-specific promoter, is in the recruiting stage (Table 1). Up to three nominal dose levels of AT845 have been planned to be evaluated in this study. A single AT845 administration through intravenous infusion will be performed. The initial dose cohort will receive a single dose of 3 × 10^13^ vector genomes (vg)/kg, the second dose cohort will receive a single dose of 6 × 10^13^ vg/kg, and the third dose cohort, a single dose of 1 × 10^14^ vg/kg. Subjects will be observed for the safety and efficacy of AT845 over 48 weeks.

In the NCT 00976352 study, all subjects required chronic and full-time mechanical ventilation owing to respiratory failure and were unresponsive to both ERT and preoperative muscle-conditioning exercises. After receiving a dose of either 1 × 10^12^ vg (*n* = 3) or 5 × 10^12^ vg (*n* = 2) of rAAV1-h*GAA* vector, the subjects’ unassisted tidal volume increased significantly [median (interquartile range) 28.8% increase (15.2–35.2), *p* < 0.05]. Further, most patients tolerated appreciably longer periods of unassisted breathing [425% increase (103–851), *p* = 0.08]. Gene therapy did not contribute to improving maximal inspiratory pressure. No T-cell-mediated immune responses to the vector and expected levels of circulating antibodies were detected. The results support the safety and moderate efficacy of rAAV1-h*GAA* (Smith et al., 2013). In the NCT 02240407 study, the potential activity, biodistribution, and toxicology of the re-administration of rAAV9-desmin enhancer/promoter-human *GAA* vector injected intramuscularly into the tibialis anterior were investigated in two patients with LOPD.

ACTUS-101 is an AAV 8 vector encoding human GAA under the control of the light strand promoter. Liver depot gene therapy is considered to be an effective strategy for treating PD (Kishnani and Koeberl, 2019). Ding et al. (2002) reported the presence of the high molecular weight precursor AαGlu in the blood, which was taken up and processed to mature AαGlu in skeletal and heart muscle where accumulated lysosomal glycogen was disassembled effectively. Although *GAA* expression in the liver proved to be transient, adenovirus vector-mediated *GAA* expression from the liver depot achieved a high level of biochemical correction in the skeletal and heart muscle (Ding et al., 2002).

In PD gene therapy, the administration is intravenous or intramuscular. Both intravenous and intramuscular administration of AAV vector encoding h*GAA* is expected to be effective. In the future, the better-suited administration route for the AAV vector may be determined based on a patient’s disease type, IOPD or LOPD, as the target organ for gene therapy varies depending on disease type and severity.

### 2.3 Gaucher disease

Gaucher disease (GD, MIM: 606463) is an autosomal recessive LSD caused by mutations in the glucocerebrosidase (*GBA*) gene encoding glucocerebrosidase (GCase, EC: 3.2.1.45). GCase catalyzes the conversion of glucosylceramide to ceramide (Figure 1). Defective GCase leads to the accumulation of glucosylceramide and/or glucosylsphingosine in the lysosomes of various cells, resulting in systemic manifestations of GD, including bone and neural impairments, hematological defects, and hepatosplenomegaly (Platt, 2014).

In the NCT00001234 study, the safety and feasibility of retroviral transduction of peripheral blood or bone marrow CD 34 + cells with *GBA* cDNA were investigated. Unfortunately, the level of corrected cells (<0.02%) was too low to induce any clinical benefit, and GCase activity did not increase in any patient (*n* = 3) following the infusion of transduced cells (Table 1) (Dunbar et al., 1998).

AVR-RD-02 is a drug comprising autologous CD34+ enriched HSCs genetically modified *ex vivo* using an LV to contain an RNA transcript that results in a codon-optimized cDNA encoding human *GBA*. In GD type I model mice, AVR-RD-02 led to a significant decrease in glucocerebroside accumulation with subsequent improvement of hepatosplenomegaly, restoration of blood parameters, and a tendency for enhanced bone mass and density. Moreover, a significant reduction in Gaucher cells in the bone marrow was seen after gene therapy (Table 1) (Dahl et al., 2021). In NCT 04145037, the efficacy and safety of AVR-RD-02 will be assessed in 8–16 subjects (male and female) between the ages of 18 and 50 years old with GD Type I. After AVR-RD-02 transplant, the subjects will enter the post-transplant follow-up period (∼52 weeks). During this time, periodic efficacy and safety assessments will be performed to assess measures of safety, engraftment, and clinical response post-transplant. Moreover, subjects will be followed up for an additional 14 years to monitor safety and changes in selected biomarkers and clinical outcomes (NCT04836377).

PR001 (LY3884961) is an AAV9 vector encoding human *GBA*. In NCT 04411654, the efficacy and safety of a single dose of PR001 will be evaluated in infants with GD Type II. Subjects will be investigated for PR001 efficacy, safety, tolerability, biomarkers, and immunogenicity in the first 12 months, and followed up for an additional 4 years to monitor safety and changes in selected biomarkers and clinical outcomes.

Liver-targeted (*in vivo*), brain-targeted (*in vivo*), and HSC-targeted (*ex vivo*) clinical trials of GD are ongoing. In liver-targeted *in vivo* therapy, a hepatotropic AAVS3 vector is used. The transfer of the *GBA* gene to hepatocytes is expected to produce GCase enzymes and express therapeutic effects. In brain-targeted *in vivo* therapy, the AAV9 vector is used. AAV9/*GBA* intracisternal administration may transduce oligodendrocytes to produce GBA and express therapeutic effects. In *ex vivo* therapy, the *GBA* gene is transduced to patients’ CD34+ HSCs using LV or RV. The transduced HSCs will deliver the functional enzyme to sites not accessible by ERT. In the future, the therapeutic strategy may be determined based on a patient’s disease type and severity.

### 2.4 Krabbe disease

Krabbe disease (KD; globoid cell leukodystrophy) (MIM: 245200) is an autosomal recessive LSD caused by a galactosylceramide-β-galactosidase (GALC, EC: 3.2.1.46) defect. Both psychosine and galactosylceramide accumulate in the white matter of the brain. The infantile-onset KD may be manifested as hypersensitivity to external stimuli, irritability, and severe mental and motor deterioration, which usually appears before 6 months of age. Most patients die before their second birthday without appropriate treatment. Patients categorized as an attenuated phenotype often develop symptoms later in life (Ferreira and Gahl, 2017). The main pathology of KD is the demyelination of neurons in the brain white matter; thus, myelin regeneration is expected to be a potential treatment. HSC transplantation, which can promote central myelination, is an effective treatment option for newborn and infantile patients before the start of neurodegeneration (Allewelt et al., 2018).

PBKR03 is an AAV vector serotype Hu68 carrying human *GALC* cDNA. NCT 04771416 is a study of PBKR03 delivered as a one-time dose administered into the cisterna magna of subjects with early infantile KD. Hordeaux et al. (2022) reported the safety, scalability, and efficacy of a single cisterna magna PBKR03 administration to treat KD model mice and dogs (Table 1).

FBX-101 is a replication-deficient AAV vector encoding human *GALC*, which is delivered one time through a venous catheter inserted into a peripheral limb vein. NCT 04693598 is a non-randomized, non-blinded, dose escalation study of intravenous AAVrh10 after HSC transplantation. Bradbury et al. (2018) reported the effect of AAV gene therapy in a canine model of KD, which recapitulated human clinical manifestations. A combination of intracerebroventricular and intravenous injections of AAVrh10 therapy, targeting both the central and peripheral nervous systems, had a clear dose response and led to delayed onset of clinical signs, correction of biochemical defects, attenuation of neuropathology, and extended life-span (Table 1).

The target organ for KD gene therapy is the brain because the neurodevelopmental outcome is crucial. The intracerebroventricular administration of the AAV vector is expected to be useful in the early treatment of infants before the presentation of neurological manifestations.

### 2.5 Mucopolysaccharidoses

Mucopolysaccharidoses (MPSs) are a heterogeneous group of LSDs, clinically manifested by reduced life expectancy and progressive dysfunction in multiple organs (Coutinho et al., 2012). Mucopolysaccharides are complex sugar molecules identified in connective tissues and other tissues throughout the body, including the liver, spleen, cartilage, skin, cornea, and vascular tissue. MPS I (Hurler Syndrome, MIM: 607014), II (Hunter syndrome, 309900), IIIA (Sanfilippo syndrome A, 252900), IIIB (Sanfilippo syndrome B, 252920), IIIC (Sanfilippo syndrome C, 252930), IIID (Sanfilippo syndrome D, 252940), IVA (Morquio syndrome A, 253000), IVB (Morquio syndrome B, 253010), VI (Maroteaux-Lamy syndrome, 253200), and VII (Sly syndrome, 253220) are caused by a defect in *α*-L-iduronidase (IDUA, EC: 3.2.1.76), iduronate-2-sulfatase (IDS, EC: 3.1.6.13), heparan-N-sulfatase (SGSH, EC: 3.10.1.1), N-acetylglucosaminidase (NAGLU, EC: 3.2.1.50), heparan-alpha-glucosaminide N-acetyltransferase (HGSNAT, EC: 2.3.1.78), N-acetylglucosamine-6-sulfatase (GNS, EC: 3.1.6.14), N-acetylgalactosamine-6-sulfate sulfatase (GALNS, EC: 3.1.6.4), *β*-galactosidase (*GLB1*, EC: 3.2.1.23), arylsulfatase B (*ARSB*, EC: 3.1.6.12), and *β*-glucuronidase (*GUSB*, EC: 3.2.1.31), respectively. Patients with MPS are generally healthy at birth, but present clinical manifestations, including deterioration of the joints, airways, and skeletal muscle; impaired vision and hearing; and heart valve disease, from early childhood. Most patients with MPS I, II, III, or VII also develop impaired cognitive ability.

NCT 02702115, 04628871, 03580083, and 03488394 studies of gene therapy for MPS I, and NCT 00004454, 04571970, 03566043, 04597385, 03041324, 04628871, and 05238324 studies of gene therapy for MPS II have been carried out. NCT 01474343 and 02053064 studies of gene therapy for MPS ⅢA are completed, NCT 04088734 was terminated, and NCT 03612869, 02716246, 04360265, 04201405, and EudraCT 2015-000359-26 are underway. NCT 03300453 study of gene therapy for MPS ⅢB is completed, and NCT 03315182 and 04655911 are underway. For MPS Ⅵ, NCT 03173521 is underway (Table 1).

SB-318 is a zinc finger nuclease (ZFN)-mediated *in vivo* genome editing tool used to insert a normal copy of the *IDUA* transgene into liver cells, delivered *via* AAV2/6 vectors. Ou et al. (2020) developed a proprietary system gene editing approach using CRISPR, which improves transduction efficacy and involves the insertion of a promoter-less human *IDUA* cDNA sequence into the albumin locus of hepatocytes. Gene therapy was performed in neonatal and adult MPS Ⅰ mice and led to a significant increase in IDUA enzyme activity in the brain and a decrease in the content of storage substrates. Furthermore, the treated mice acquired improved memory and learning ability. No vector-associated toxicity or increased tumorigenesis risk was observed (Ou et al., 2020). Harmatz et al. (2022) reported the result of a phase 1/2 clinical trial for 3 adult patients with attenuated MPS Ⅰ. The administration of SB-318 once intravenously at a dose of 1 × 10^13^ vg/kg (n = 1) and 5 × 10^13^ vg/kg (n = 2) showed a good safety profile and evidence of targeted genome editing in the liver up to 48 weeks after administration.

Gentner et al. (2021) reported the result of NCT 03488394. They administered autologous HSCs transduced *ex vivo* with LV encoding human *IDUA* in subjects with MPS Ⅰ after myeloablative conditioning. All of the subjects exhibited sustained supraphysiologic blood IDUA activity. Urinary glycosaminoglycan (GAG) excretion significantly decreased to normal levels following treatment. The cerebrospinal fluid IDUA activity was increased after gene therapy and was correlated with local clearance of GAGs. Treated subjects demonstrated stable cognitive ability and motor skills, reduced joint stiffness, and normal growth.

SB-913 is a ZFN-mediated *in vivo* genome editing tool used to insert a normal copy of the *IDS* transgene into liver cells, delivered *via* AAV2/6 vectors. Harmatz et al. (2022) reported the result of a phase 1/2 clinical trial for nine adult patients with attenuated MPS Ⅱ. The administration of SB-913 once intravenously at a dose of 5 × 10^12^ vg/kg (n = 2), 1 × 10^13^ vg/kg (n = 2), and 5 × 10^13^ vg/kg (n = 5) showed a good safety profile and evidence of targeted genome editing in the liver up to 48 weeks after administration.

Tardieu et al. (2014) reported the results of the intracerebral administration of AAV rh.10 carrying human *SGSH* and *SUMF1* cDNAs in four children with MPS ⅢA. The vectors were delivered bilaterally to the white matter, anterior, medial, and posterior to the basal ganglia. Three patients were able to walk, but their cognitive abilities were abnormal and had declined with brain atrophy. Treatment resulted in moderate improvement in sleep, attention, and behavior in three patients. Fu et al. (2016) treated MPS ⅢA mice at the age of 1, 2, 3, 6, and 9 months with an intravenous injection (1 × 10^12^ vg/kg) of scAAV9-U1a-hSGSH vector, resulting in the reduction in GAG levels and restoration of SGSH activity throughout the CNS and somatic tissues. Treatment up to 3 months of age improved cognitive ability in the Morris water maze at 7.5 months, and lifespan was normalized. In mice treated at 6 months of age, behavioral performance was impaired at 7.5 months, but it did not decline further when retested at 12 months, and lifespan was extended, but not normalized. Treatment at 9 months of age did not extend lifespan, although the GAG storage pathology in the CNS was alleviated. Gene therapy may be effective in the early stage of the disease. Ellison et al. (2019) optimized hCD34+ cell transduction with a clinical grade *SGSH* vector, providing improved pharmacodynamics and cell viability, and validated an effective scale-up and cryopreservation method to generate an investigational medicinal product. They also established the pre-clinical efficacy and safety of transgene HSC transplantation for MPS ⅢA mice.

Tardieu et al. (2017) reported the results of phase 1/2 clinical trials of intracerebral administration of AAV2/5 carrying human *NAGLU* cDNAs for four children with MPS ⅢB. No unexpected significant adverse drug reactions were observed. Sustained enzyme production was induced in the brain, and neurocognitive development progressed in all patients, with the youngest patient having neurological function close to that seen in healthy children. Gougeon et al. (2021) performed a comprehensive analysis of cytokine patterns and cellular immunity in the four treated children over 5.5 years of follow-up. Memory and polyfunctional CD8+ and CD4+ T lymphocytes, which were sensitized to the transgene, began appearing soon after the start of treatment in peripheral blood and waves throughout the follow-up. However, this response had no apparent impact on CNS transgene expression, which remained stable 66 months after surgery. Gene therapy did not trigger neuroinflammation through the expression of chemokines and cytokines in the CSF of the patients. Ribera et al. (2015) administered AAV9 vectors encoding murine *NAGLU* cDNA, under the control of the chicken β-actin/cytomegalovirus (CMV) enhancer ubiquitous promoter, to cisterna magna of 2-month-old male and female MPS IIIB mice. Restoration of enzymatic activity in the CNS led to the normalization of GAG content and lysosomal physiology, alleviated neuroinflammation, and restored the pattern of gene expression in the brain similar to that of healthy animals. Additionally, transduction in the liver, owing to the passage of vectors into the circulatory system, led to whole-body disease correction. Treated animals also showed improvement in behavioral deficits and an extended lifespan.

As gene therapy for MPS IIID, Roca et al. (2017) administered 5 × 10^10^ vg of AAV9 vector expressing murine *Gns* into 2-month-old *Gns* ^−/−^ mice through cisterna magna injection. The treatment with AAV9-*Gns* corrected pathological storage, improved lysosomal functionality in the CNS and somatic tissues, resolved neuroinflammation, restored normal behaviour and extended lifespan of treated mice (Table 2).

As gene therapy for MPS ⅣA, Sawamoto et al. (2020) administered AAV8 vector expressing different forms of human *GALNS*, under a liver-specific promoter, intravenously into 4-week-old MPS ⅣA mice. GALNS enzyme activity was significantly enhanced in the plasma of all treated mice at 2 weeks post-injection and was maintained throughout the monitoring period. Treatment with AAV vectors led to a decrease in keratan sulfate levels in plasma to normal levels at 2 weeks after injection, which was maintained until necropsy. Gene therapy decreased the storage of keratan sulfate in ligaments, articular cartilage, and meniscus surrounding articular cartilage and growth plate regions, as well as, heart muscle and valves. This result suggests that AAV gene therapy is a novel treatment to address heart and bone disease in patients with MPS ⅣA (Table 2).

Ferla et al. (2017) showed that a single systemic administration of a recombinant AAV2/8 vector encoding *ARSB*, under the transcriptional control of the liver-specific thyroxine-binding globulin promoter (AAV2/8. TBG.hARSB), led to sustained liver transduction and phenotypic improvement in MPS Ⅵ mice. Moreover, they tested both the efficacy and safety of a single intravenous administration of AAV2/8. TBG.hARSB at doses ranging between 2 × 10^11^ and 2 × 10^12^ genome copies/kg body weight. AAV-treated mice showed no toxicity except for thyroid epithelial hypertrophy and a transient increase in alanine aminotransferase activity in females. AAV2/8. TBG.hARSB expression and biodistribution were confirmed in the liver as the main site of both transduction and infection. The risk of both horizontal and germline transmission was minimal (Table 1).

Macsai et al. (2012) injected an LV vector encoding the murine β-glucuronidase gene, under the transcriptional control of the elongation 1 alpha promoter, intravenously into MPS Ⅶ mice at birth or 7 weeks. High levels of vector expression were demonstrated in the spleen, liver, and bone marrow, and to a lesser extent in the lung, heart, and kidney after injection. Widespread clearance of GAG storage was detected in somatic tissues of both groups, and some clearance of neuronal storage was observed in mice treated at birth. Therefore, intravenous LV gene therapy results in a measurable improvement in parameters of bone mass and architecture, as well as enzymatic and biochemical correction. In contrast, growth plate chondrocytes are non-responsive to therapy.

Gene therapies for neuronopathic MPS are progressing and many clinical trials are ongoing (Table 1). There are two types of approaches for targeting the CNS: the direct transfer of a therapeutic gene into the brain (*in vivo*) and HSC-targeted gene therapy (*ex vivo*). To date, AAVs are the most used viral vectors in gene therapy, followed by *ex vivo* gene therapy with lentiviral vectors. Although encouraging results are reported, several challenges remain to be overcome in vector selection, administration route, and immunogenicity. Gene editing technology has recently also been applied for MPS I, MPS II, and hemophilia B in human (Harmatz et al., 2022). It is extremely promising that these first-in-human trials proven *in vivo* genome editing.

### 2.6 GM1 gangliosidosis

GM1 gangliosidosis (MIM: 230600) is an autosomal recessive LSD caused by a β-galactosidase (βG, EC: 3.2.1.23) defect due to a *GLB1* gene mutation (Figure 1). The defect leads to the accumulation of GM1 ganglioside in various tissues, as the enzyme degrades GM1 to GM2 gangliosides, especially in the peripheral nervous system and CNS (Sandhoff and Harzer, 2013). The major clinical manifestations of GM1 gangliosidosis are neural impairment, such as seizure and motor and cognitive developmental delays (Brunetti-Pierri and Scaglia, 2008).

NCT 03952637 is a phase 1/2 study aimed at assessing the safety and efficacy of the AAV9-*GLB1* vector following intravenous delivery. Latour et al. (2019) demonstrated that the microinjection of AAV9-*GLB1* vector into *GLB1* knockout organoids showing a progressive accumulation of GM1 ganglioside significantly enhanced *β*-gal activity and significantly reduced GM1 ganglioside. Subsequently, clinical trials using the AAV9-*GLB1* vector were conducted (Table 1).

LYS-GM101 is an AAVrh.10 vector carrying human *GLB1* cDNA. In NCT 04273269, the safety and efficacy of intracisternal administration of LYS-GM101 in subjects with infantile GM1 gangliosidosis were evaluated. PBGM01 is an AAVHu68 vector carrying *GLB1* cDNA. The efficacy, tolerability, and safety of a single dose of PBGM01 administered *via* the intra-cisterna magna were investigated in infantile patients with GM1 gangliosidosis in NCT 04713475 (Table 1).

GM1 gangliosidosis significantly impacts the CNS. Intracranial administration of AAV vector encoding *hGLB1* in early infants with GM1 gangliosidosis may lead to better neurodevelopmental outcomes; no other administration route other than intracranial is being tested for AAV vector administration.

### 2.7 Tay-Sachs/Sandhoff disease

GM2 gangliosidosis is a heterogeneous autosomal recessive LSD caused by a β-hexosaminidase (EC: 3.2.1.52) defect (Figure 1). The causative mutations in different genes may lead to three types of diseases, namely, Sandhoff-Jatzkewitz disease (SD; MIM: 268800), Tay-Sachs disease (TSD; MIM: 272800), and GM2 ganglioside activator protein deficiency (MIM: 272750). There are two isoenzymes of β-hexosaminidase: HEXA consisting of α and β subunits and HEXB, a homodimer of β subunits. The clinical phenotypes of GM2 gangliosidosis vary widely and are characterized by a neurological disease that manifests as spinocerebellar and motor dysfunction.

AXO-AAV-GM2 is aimed at restoring HEXA function by introducing a functional copy of the *HEXA* and *HEXB* genes *via* co-administration of two vectors based on the neurotropic AAVrh.8 vector carrying human *HEXA* or *HEXB* cDNA. In NCT 04669535, the bilateral intraparenchymal thalamic and intracisternal/intrathecal administration of AXO-AAV-GM2 in TSD or SD is being studied. Flotte et al. (2022) reported the results of an expanded-access clinical trial in two patients with infantile TSD (IND 18225). The patients were treated with an equimolar mix of AAVrh8-*HEXA* and AAVrh8-*HEXB* administered intrathecally at a total dose of 1 × 10^14^ vg. No vector-related adverse events were observed. HexA activity in cerebrospinal fluid increased and remained stable in both patients. This study provides early safety and proof-of-concept data for the treatment of patients with TSD using AAV gene therapy.

TSHA-101 is an AAV9 vector containing *HEXA* and *HEXB* cDNA. NCT 04798235 is aimed at investigating the safety and tolerability of intrathecally administered TSHA-101 (Table 1).

The most common symptoms of GM2 gangliosidosis are neurologic involvement. Therefore, targeting the CNS is the priority of GM2 gangliosidosis gene therapy. Two candidates, both base on neurotropic AAV, are being clinically tested *via* intracisternal/intrathecal administration. The first-in-human trials are promising, and long-term safety and efficacy data are soon expected.

### 2.8 Cystinosis

Cystinosis is an autosomal recessive LSD caused by mutations in the *CTNS* gene encoding the carrier protein cystinosin that transports cystine out of the lysosomal compartment. A defect in cystinosin function leads to the accumulation of lysosomal cystine throughout the body. The kidneys are often affected during the first year of life by proximal tubular damage followed by progressive glomerular disorder and renal failure during mid-childhood. This disease has three major clinical manifestations depending on the severity of *CTNS* gene damage: The infantile nephropathic form (MIM: 219800, ORPHA411629), the juvenile nephropathic form (MIM: 219900, ORPHA 411634), and the ocular non-nephropathic form (MIM: 219750, ORPHA411641) (Gahl et al., 2000).

CTNS-RD-04 and AVR-RD-04 are peripheral blood autologous CD34+ enriched cell fractions transduced with LV vector pCCL-CTNS containing human *CTNS* cDNA. The NCT 03897361 study has evaluated the safety, tolerability, and efficacy of CTNS-RD-04 in patients. Moreover, the NCT 05146830 study intends to assess the long-term safety and durability of AVR-RD-04 treatment for a total of 15 years (Table 1). Harrison et al. (2013) demonstrated the effect of genetically modified HSCs on the expression of a functional *CTNS* transgene using a self-inactivating LV vector in the cystinosis model mice. Transduced HSCs decreased cystine content in all tissues and improved kidney function (Table 1) (Harrison et al., 2013).

Gene therapy for cystinosis involves the use of autologous CD34+-enriched cell fractions transduced with LV vector encoding *hCTNS*. Although the intravenous administration of AAV vector encoding *hCTNS* gene may be effective in limited cases, such as kidney disease, one dose of autologous CD34+-enriched cell fractions transduced with LV vector encoding *hCTNS* gene is considered to have beneficial effects throughout the body, while several rounds of intravenous administration are needed.

### 2.9 Metachromatic leukodystrophy

Metachromatic leukodystrophy (MLD, MIM: 250100) is caused by the defect of arylsulfatase A (ARSA, EC: 3.1.6.1), a key enzyme in the catabolism of myelin-enriched sphingolipids (Figure 1). The progressive accumulation of sulfatides results in severe demyelination and neurodegeneration of the peripheral nervous system and CNS (Gieselmann et al., 1998).

Gene therapy clinical trials, namely, MLD, NCT 04283227, 03392987, 01560182, 01801709, 02559830, and 03725670 have been performed. Sessa et al. (2016) and Fumagalli et al. (2022) reported the results of NCT 01560182 (Table 1). Fumagalli et al. (2022). performed a phase 1/2 clinical study on 29 pediatric patients with pre-symptomatic or early-symptomatic early-onset MLD treated with arsa-cel, a gene therapy containing an autologous HSC population transduced *ex vivo* with an LV vector encoding human *ARSA* cDNA (OTL-200). ARSA activity in peripheral blood mononuclear cells in patients was significantly increased above baseline 2 years after treatment. Most patients who received *ex vivo* gene therapy progressively improved cognitive development and motor skills. Prevention or delay of brain atrophy and peripheral and central demyelination throughout follow-up were observed. Treatment effects were particularly evident in pre-symptomatic patients. All patients had at least one grade 3 or higher adverse event, most of which were related to conditioning or background disease. The only adverse event related to arsa-cel was the transient development of anti-ARSA antibodies in four patients, which did not affect clinical outcomes (Fumagalli et al., 2022). OTL-200 was approved as atidarsagene autotemcel (Libmeldy^Ⓡ^) in December 2020 by the European Medicines Agency (EMA) (Table 1).

Piguet et al. (2012) reported the short-term effects of an AAVrh.10 vector encoding human *ARSA* cDNA under the control of the cytomegalovirus/β-actin hybrid promoter in 8-month-old MLD mice that showed marked sulfatide accumulation and brain pathology. The AAVrh.10cuARSA vector improved sulfatide storage in the brain, accumulation of specific sulfatide species in oligodendrocytes, and associated brain pathology 2 months after intracerebral injection (Table 1).

MLD is caused by a deficiency of ARSA, which leads to the accumulation of sulfatides in both the CNS and peripheral nervous system. The rate of progression of symptoms in MLD is rapid and the gene therapy product become available. We should consider MLD as a candidate for newborn screening.

### 2.10 Neuronal ceroid lipofuscinosis

Neuronal ceroid lipofuscinoses (NCLs) are a group of extremely rare and fatal neurodegenerative LSDs caused by mutations in at least 14 different genes (CLN1–CLN14). They are manifested by the intracellular accumulation of autofluorescent lipofuscin and fatty lipopigment in both peripheral tissues and the brain (Mole and Cotman, 2015).

NCT 02725580, 01414985, 01161576, and 00151216 studies have been completed in late-infantile NCL (LINCL) patients (Table 1). Worgall et al. (2008) administered a total average dose of 2.5 × 10^12^ particle units of an AAV2 vector encoding human CLN2 cDNA (AAV2CUh-CLN2) into 12 locations in the CNS in 10 children with LINCL. The rate of neurological decline in the treated participants at 18 months was evaluated using a neurological rating scale and three quantitative magnetic resonance imaging parameters. Although four participants exhibited a transient, mild, humoral response to the vector, AAV2CUh-CLN2 administration contributed to a significantly reduced rate of neurological decline.

The safety of the administration of gene therapy for CLN3, CLN5, or CLN7, an AAV9 vector encoding human *CLN3*, *CLN5*, or *CLN7* cDNA, respectively, has been evaluated in clinical trials (Table 1). Clinical trials for CLN1, CLN8, and CLN10 are soon expected considering the positive effect of gene therapy on disease model mice (Table 2).

For CLN1, Shyng et al. (2017) developed an AAV2/9 vector containing cDNA encoding the lysosomal enzyme palmitoyl protein thioesterase 1 (PPT1). This vector was targeted to the spinal cord *via* intrathecal administration in Ppt1^−/−^ mice, whereby it significantly improved motor function and life span. For CLN8, Johnson et al. (2021) developed an AAV9 vector encoding human *CLN8* cDNA under the control of the MecP2 promoter (AAV9. pT-MecP2. CLN8). A single neonatal intracerebroventricular injection of AAV9. pT-MecP2. CLN8 in motor neuron degeneration mice led to the expression of the transgene in the CNS from 4 to 24 months, alleviating behavioral and histopathological hallmarks and improving survival duration. Moreover, the performance of the motor neuron function test improved (Johnson et al., 2021). For CLN10, Shevtsova et al. (2010) developed an AAV1/2 vector encoding mouse cathepsin D (CtsD) under the control of the CMV/human β-actin hybrid promoter (AAV1/2-CtsD). Injection of AAV1/2-CtsD into the brain of CtsD^−/−^ mice resulted in extended survival duration and prevented both central and visceral pathologies. CtsD is secreted from CNS neurons and drained from the CNS to the periphery *via* lymphatic routes. Therefore, CtsD acts as an important modulator of peripheral tissue homeostasis and immune system maintenance (Shevtsova et al., 2010).

NCLs are CNS neurodegenerative LSDs; all clinical trials of NCL gene therapy have been performed using intracranially or intrathecally administered AAV vector. Although results so far are encouraging, the effect of NCL gene therapy in the CNS remains to be elucidated.

### 2.11 Danon disease

Danon disease (MIM: 300257) is an X-linked disorder caused by the deficiency of lysosomal-associated membrane protein (LAMP2). It is manifested by skeletal myopathy, hypertrophic cardiomyopathy, and impaired intelligence in male patients, and by a milder phenotype involving cardiac muscle in female patients (Boucek et al., 2011). In NCT 03882437, a phase 1 study to evaluate the toxicity and safety of gene therapy using a recombinant AAV9 vector containing the human *LAMP2* isoform B transgene (RP-A501) was performed in male patients with Danon disease (Table 1).

Rocket Pharmaceutocals Inc. Announced the phase 1 data of RP-A501 on 30 September 2022. Seven patients were intravenously administrated low-dose (6.7 × 10^13^ gc/kg, n = 5) or high-dose (6.7 × 10^14^ gc/kg, n = 2) of RP-A501; RP-A501 was well tolerated and improved cardiac symptoms and vacuole clearance and led to marked reductions in brain natriuretic peptide and troponin. The first-in-human trials are promising; the long-term safety and efficacy data are soon expected.

### 2.12 Aspartylglucosaminuria

Aspartylglucosaminuria (AGU) (MIM: 208400) is an inherited recessive and neurodegenerative LSD caused by mutations in the aspartylglucosaminidase gene (*AGA*), resulting in glycoasparagine accumulation and cellular dysfunction. This disease is manifested by skeletal abnormalities, connective tissue overgrowth, gait disturbance, progressive intellectual disability, and seizures followed by premature death.

Chen et al. (2021) intravenously or intrathecally administered a recombinant AAV9 vector encoding human *AGA* c DNA (AAV9/*AGA*) in *Aga* ^−/−^ mice before or after disease onset (Table 2). At each treatment age, AAV9/*AGA* administration achieved 1) a dose-dependent increase and sustained AGA activity in body fluids and tissues; 2) a rapid, sustained, and dose-dependent elimination of AGA substrate in body fluids; 3) significantly improved locomotor activity; 4) dose-dependent prevention of loss of Purkinje neurons in the cerebellum; 5) significantly ameliorated gliosis in the brain. Moreover, treated mice presented no abnormal neurological phenotype and maintained body weight throughout the experiment until 18 months of age.

In the future, clinical trials of AGU gene therapy using AAV/*AGA* are expected.

### 2.13 Niemann-Pick C

Niemann-Pick disease type C (NPC, MIM: 257220) is caused by mutations in either *NPC1* or *NPC2* that encode proteins involved in the transportation of free cholesterol from lysosomes to the endoplasmic reticulum (ER). Defects in *NPC1* or *NPC2* result in abnormal lipid storage of free cholesterol and GM1 gangliosides in the spleen, liver, and CNS (Dahl et al., 2021). The major hallmarks of NPC are neural impairment, including cerebellar ataxia, gait problems, dysarthria, seizures, and developmental delay. Patients also develop liver dysfunction and hepatosplenomegaly (Patterson et al., 2012).

Jiang et al. (2020) developed gene therapy for NPC1 using Trojan horse liposomes (THLs), which were encapsulated with 8.0 kb of plasmid DNA encoding the 3.9 kb human *NPC1* open reading frame, under the influence of a 1.5 kb platelet derived growth factor B promoter. This liposome was administered weekly beginning at 6–7 weeks to *Npc*1^−/−^ null mice. Delivery of the plasmid DNA and NPC1 mRNA expression in the spleen, liver, and brain were observed. THLs treatment alleviated tissue inclusion bodies in the peripheral organs and brain but did not extend the lifespan in NPC model mice (Table 2) (Jiang et al., 2020). Kurokawa et al. (2021) also developed an AAV9/3 vector encoding human *NPC1* cDNA under a CMV promoter (AAV-CMV-hNPC1), and administered it into the cisterna magna and left lateral ventricle of *Npc1* ^−/−^ mice at an age of 4 or 5 days. AAV-CMV-hNPC1 therapy improved the survival duration, body weight, and rotarod test performance in *Npc1* ^−/−^ mice (Table 2). Moreover, cerebellar Purkinje cells were also preserved in AAV-CMV-hNPC1 treated *Npc1* ^−/−^ mice at 11 weeks. Injection into both the cisterna magna and lateral ventricle contributed to delivering AAV-CMV-hNPC1 into body tissue including the brain, liver, lung, and heart.

The preclinical data are promising, and clinical trials of NPC gene therapy are soon expected.

### 2.14 Alfa mannosidosis

Alpha-mannosidosis (AM) (MIM: 248500) is an autosomal recessive LSD, caused by a lysosomal enzyme alpha-mannosidase defect due to pathogenic variants in the *MAN2B1* gene. AM is characterized by the accumulation of mannose-rich oligosaccharides, which causes apoptosis and impaired cellular function, and results in diverse adverse clinical manifestations (Beck et al., 2013).

Yoon et al. (2020) administered an AAVhu.32 vector encoding human *MAN2B1* cDNA (AAVhu.32-MAN2B1) into the carotid artery of AM cat model 4–6 weeks of age (Table 2). The vector was distributed throughout the brain from the olfactory bulb to the rostral end of the cerebellum, predominantly in the grey matter. The AAVhu.32-MAN2B1 vector ameliorated the severity of neurological manifestations and extended the survival of cats with AM disease. The extent of therapy was dose-dependent. Diffusion tensor imaging and magnetic resonance spectroscopy demonstrated differences between the effects of high and low doses, and normalization of grey and white matter imaging parameters at the higher dose (Yoon et al., 2020). Although AM is a CNS neurodegenerative disease, intravenous high-dose administration of AAV vector may lead to amelioration of clinical manifestation and is expected to be a promising therapeutic approach.

### 2.15 Galactosialidosis

Galactosialidosis (GS) (MIM: 256540) is a glycoprotein storage disease caused by mutations in *CTSA*, encoding the lysosomal carboxypeptidase protective protein cathepsin A. GS affects different body systems, including the eyes, brain, skeleton, and muscles and develops manifestations, including vision problems, difficulty walking, dark red spots on the skin, spine abnormalities, and intellectual disability, that gets worse with time (Caciotti et al., 2013). Manifestations of the early and late infantile types are severe and associated with decreased survival. Hu et al. (2021) reported a long-term safety and efficacy preclinical study for GS mice (Table 2). One-month-old *Ctsa* ^−/−^ mice were injected intravenously with a high dose of a self-complementary AAV2/8 vector expressing human *CTSA* in the liver. Treated GS mice, which were examined up to 12 months after receiving gene therapy, appeared indistinguishable from their wild-type mice. Sustained expression of scAAV2/8-CTSA in the liver led to the release of the therapeutic precursor protein in circulation and its widespread uptake by cells in the brain and visceral organs. Enhanced cathepsin A activity improved lysosomal vacuolation throughout the affected organs and sialyl-oligosacchariduria (Hu et al., 2021).

Clinical trials of GS gene therapy using AAV/*CTSA* are expected in the future.

### 2.16 Mucolipidosis IV

Mucolipidosis type Ⅳ (ML Ⅳ) (MIM: 252650) is an LSD caused by mutations in *MCOLN1*. Patients with ML Ⅳ show delayed developmental milestones in infancy and reach a plateau in psychomotor development by 2 years of age. Signs of extrapyramidal and pyramidal motor dysfunction and axial hypotonia are manifested early in life. Patients suffer from impaired independent ambulation and severely limited fine motor function. Moreover, patients frequently exhibit progressive spastic quadriplegia across the first decade of life with significant loss of gross and fine motor skills during the second decade (Wakabayashi et al., 2011).

In a study by DeRosa et al. (2021), AAV-mediated CNS-targeted gene transfer of human *MCOLN1* restored motor function and alleviated brain pathology in ML Ⅳ mice (Table 2). The vector administration in symptomatic ML Ⅳ mice resulted in long-term reversal of declined motor function and a delay in paralysis. Intracerebroventricular administration of a self-complementary AAV9 clinical candidate vector in post-natal day 1 mice significantly restored motor function and myelination and reduced lysosomal storage load in the ML Ⅳ mouse brain. The scAAV9-mediated CSF-targeted *MCOLN1* transfer is a potential therapeutic strategy for ML Ⅳ (DeRosa et al., 2021).

In the future, gene therapy for Mucolipidosis IV may be an effective treatment approach.

### 2.17 Farber disease

Farber disease (MIM: 228000) is an autosomal recessive LSD caused by an acid ceramidase (AC; EC 3.5.1.23) activity defect (Figure 1) (Sugita et al., 1972). AC activity deficiency results in ceramide accumulation in various tissues, including the lung, spleen, and liver. Farber disease is characterized by painful and progressively deformed joints, subcutaneous nodules, hoarseness due to laryngeal involvement, and premature death. Moreover, hepatosplenomegaly and nervous system dysfunctions may develop. Alayoubi et al. (2013) demonstrated the effect of gene therapy in Asah1^P361R/P361R^ mice with a defect in AC, which developed Farber disease manifestations and died within 7–13 weeks (Table 2). Intravenous injection of a recombinant LV encoding human AC in Asah1^P361R/P361R^ neonates ameliorated the disease phenotype, including reduced ceramide levels, lessened cellular infiltrations, enhanced growth, and increased lifespans.

In the future, clinical trials of Farber disease gene therapy are expected.

## 3 Future gene therapy prospects for LSDs

Several clinical trials of LSD gene therapy are underway as phase 1/2 studies, and some have been completed. Most trials have not reported any adverse events resulting in their discontinuation. Moreover, gene therapies for LSDs have been adopted; for example, OLT-200 (Libmeldy^Ⓡ^) has been approved for gene therapy for MLD by the European Medicines Agency. Some clinical trial reviews on gene therapy for LSDs have been published recently (Consiglieri et al., 2022; Rajan and Escolar, 2022; Wood and Bigger, 2022). Clinical trials involving the administration of viral vectors in animal models for LSDs have achieved good therapeutic outcomes. Therefore, positive results from human clinical phase 1/2 studies using these vectors are expected in the future, which may promote the practical use of gene therapy for LSDs. Several challenges including suitable vector selection, administration route, and immunogenicity, are yet to be overcome. In particular, in CNS neurodegenerative LSDs, the selection of an ideal vector, administration route, and time is important. Only limited AAV vectors can pass through the BBB (Fu and McCarty, 2016). Therefore, gene therapy strategy should be considered depending on the target organ and the onset time.

Newborn screening for several LSDs, including FD, PD, and GD, has been conducted in Japan and worldwide. Newborn screening contributed to the identification of patients with LSDs before developing disease signs or symptoms (Sawada et al., 2020a; Sawada et al., 2020b; Sawada et al., 2021; Sawada et al., 2022). ERT, which can be administered from the neonatal period, is available for some LSDs. ERT does not affect the CNS owing to the BBB. Therefore, gene therapy is expected to be effective for infants with neurological LSDs, if therapy is provided shortly after birth, before the presentation of CNS symptoms. Moreover, ERT may be practical, especially in infants, as a bridge therapy to gene therapy. If the patient’s general condition is poor, it is difficult to perform gene therapy. ERT can be effective in maintaining a patient’s general condition. In Japan, it takes 2 weeks for onasemnogen abeparvovec (ZOLGENSMA^Ⓡ^), a gene therapy product for spinal muscular atrophy, to arrive after it is ordered. Because a considerable time elapses between diagnosis and the ability to perform genetic treatment, ERT may also be practical as a bridging therapy during that period. Therefore, new treatments such as gene therapy are expected to play an important role in the future; current treatments may also play a beneficial role depending on the status of patients with LSDs.

In conclusion, clinical gene therapy is a potential therapy for LSDs in the future. It is necessary to develop new treatment strategies, as well as, integrate the advantages of current treatments with gene therapy.

## Glossary

AAV: adeno-associated viral
AC: acid ceramidase
AGU: aspartylglucosaminuria
AM: alpha-mannosidosis
BBB: blood-brain barrier
CtsD: cathepsin D
CMV: cytomegalovirus
CNS: central nervous system
FD: Fabry disease
GAG: glycosaminoglycans
GALC: galactosylceramide-β-galactosidase
GCase: glucocerebrosidase
GD: Gaucher disease
GLA: α-galactosidase A
GS: galactosialidosis
HSC: hematopoietic stem cell
IOPD: infantile-onset PD
KD: Krabbe disease
LAMP2: lysosomal-associated membrane protein
LINCL: late-infantile NCL
LOPD: late-onset PD
LSD: lysosomal storage disease
LV: lentiviral
ML Ⅳ: mucolipidosis type Ⅳ
MLD: metachromatic leukodystrophy
MPSs: mucopolysaccharidoses
NCLs: neuronal ceroid lipofuscinoses;
NPC: Niemann-Pick disease type C
PD: Pompe disease
PPT1: palmitoyl protein thioesterase 1
THLs: Trojan horse liposomes
vg: vector genomes
ZFN: zinc finger nuclease
